# Age-Stratified Risk of Dementia in Parkinson's Disease: A Nationwide, Population-Based, Retrospective Cohort Study in Taiwan

**DOI:** 10.3389/fneur.2021.748096

**Published:** 2021-12-24

**Authors:** Ting-Ya Chang, Chun-Pai Yang, Yi-Huei Chen, Ching-Heng Lin, Ming-Hong Chang

**Affiliations:** ^1^Department of Neurology, Taichung Veterans General Hospital, Taichung, Taiwan; ^2^Department of Neurology, Kuang Tien General Hospital, Taichung, Taiwan; ^3^Department of Medical Education and Research, Taichung Veterans General Hospital, Taichung, Taiwan; ^4^School of Medicine, National Chung Hsing University, Taichung, Taiwan

**Keywords:** Parkinson's disease, Parkinson's disease dementia, Alzheimer-type pathology, dementia, major neurocognitive disorder, Lewy-related pathology

## Abstract

**Introduction:** Parkinson's disease (PD) manifests with dominant motor symptoms and a wide range of non-motor symptoms (NMS). Dementia is one of the most disabling and exhausting NMS throughout the clinical course. We conducted a population-based, age-stratified, retrospective cohort study to investigate the incidence rate and risk of dementia of patients with newly diagnosed PD, and linked to the clinicopathological PD subtypes.

**Methods:** Patients with newly diagnosed PD (PD group, *n* = 760) and control subjects (non-PD group, *n* = 3,034) were selected from the Taiwan's National Health Insurance Research Database from January 2001 to December 2005. The dementia incidence rate and dementia-free survival rate were calculated.

**Results:** The overall dementia incidence rate was 17.5 and 5.7 per 1,000 person-years in PD and non-PD groups, respectively. The PD group had a significantly higher overall risk of dementia than controls (*p* < 0.001). The younger PD patients had a lower dementia incidence rate than the older PD patients, but a higher dementia risk compared to the same age of controls (<60 years, adjusted HR 6.55, 95% CI 1.56–27.48, *p* = 0.010). The dementia-free survival rate was significantly lower in the PD group compared to the non-PD group during follow-up (*p* < 0.001).

**Conclusion:** In our study, the older age of onset in PD patients resulted in a higher incidence rate of dementia. In the young age of PD patients, the incidence rate of dementia was lower than the older PD patients, but the dementia risk was higher than controls of the same age. These findings possibly implied that there were different pathogenesis and pathologies causing dementia in younger and older PD patients.

## Introduction

Parkinson's disease (PD) is the secondmost common neurodegenerative disease in the world, and patients present with dominant motor symptoms and a wide range of non-motor symptoms (NMS). The main pathogenesis of PD is misfolding, aggregation, and propagation of the alpha synuclein (α-syn) protein in the central nervous system, which leads to degeneration of nigrostriatal dopaminergic neurons (1). Dementia, or major neurocognitive disorder, is one of the most disabling and exhausting NMS throughout the clinical course of PD. Cognitive decline in patients with PD has been ignored in the past. Because of improvements in medicine, PD patients have prolonged life expectancies, and we can observe the whole picture and cognitive changes of PD (2). The epidemiology of dementia associated with PD (PD-D) has been well-studied. The risk of dementia in PD is about 1.7- to 5.9-times higher compared to controls (3, 4). The incidence rate of dementia in PD was six-times higher than in controls after 5 years of follow-up (5). In the CamPaIGN cohort in the UK, 57% of patients developed cognitive deficits within 3.5 years, and the estimated dementia incidence was about 38.7 per 1,000 person-years of observation (6). In a prospective study in a Sydney cohort, 48% of PD patients developed dementia within 15 years after diagnosis, and the cumulative incidence was 83% at 20 years after the diagnosis (7, 8). The main pathology of PD-D is controversial. Cortical or limbic Lewy-related pathology (LP), coincidence Alzheimer-type pathology, and subcortical pathology are three types of pathology that might cause PD-D (9). These pathologies may result in the impaired projection of dopamine, noradrenaline, serotonin, and acetylcholine neurons to the neocortex (10). The widespread involvement of the brain and neocortical areas at Braak PD stages 5 and 6 seems to have the strongest pathological correlation with PD-D, and coexisting amyloid-β plaques and tau-containing neurofibrillary tangles may lead to a worse prognosis (11–13).

Therefore, we speculated that an older age of onset in PD patients may result in a higher risk of developing PD-D because older patients have a higher incidence of other coexisting pathologies. Conversely, the progression of clinical cognitive impairment may be more consistent with the Braak PD staging in earlier onset or typical PD patients. In this population-based retrospective cohort study, we investigated patients with newly diagnosed PD to estimate the incidence rate of dementia. We used age-stratified methodology to analyze the risk of dementia in different age subgroups and showed the development of PD-D using real-world evidence to link the results to clinicopathological PD subtypes.

## Materials and Methods

### Study Data Sources

This research used datasets from the Taiwan National Health Insurance Research Database (NHIRD), which provides a population-level data source for health care research derived from Taiwan National Health Research Insurance (NHI) program. This program covered 99.0% of the Taiwan population by 2004 and 99.5% of the population by 2010. Until 2018, up to 99.8% of Taiwan's population were enrolled under this program (14, 15). This database is comprehensive and representative of the real-world condition and is a good source for generating population-based evidences (16, 17). We conducted this research by using the Longitudinal Health Insurance Databases (LHIDs), which randomly sampled 1 million beneficiaries (*n* = 1 million) from the original NHIRD in the 2005 Registry of Beneficiaries (LHID 2005). The representativeness of LHIDs has been validated by NHRI. All diseases before 2016 were coded according to the International Classification of Diseases, Ninth Revision, Clinical Modification (ICD-9-CM). The patients' information was encrypted and the application of this database was approved by the Ethics Committee of NHRI. The study protocol complied with the Declaration of Helsinki.

### Study Design and Population

We selected subjects from LHID 2005 who were newly diagnosed with Parkinson's disease (ICD-9-CM code 332, A221) from January 2001 to December 2005. A previous validation study of a hospital administrative database reported a positive predictive value of more than 90% by using this definition of ICD-9-CM code in Taiwan (18–20). A long-term study in Taiwan revealed that most PD patients (82.9%) received their anti-parkinsonian medication in medical centers or regional hospitals, and about half of PD patients received their initial prescription from neurologists (21). To increase our diagnostic validity, the subjects were included only when the diagnosis was made in three or more consecutive outpatient visits or in one or more inpatient settings during follow-up in 1996–2011. Similar to our previous researches (22, 23), we excluded patients encoded with the diseases possibly causing secondary parkinsonism (for example: stroke, hydrocephalus, hypoxic encephalopathy…etc., see Supplementary Table 1) and excluded patients taking the medication with the side effects of extrapyramidal symptoms within 3 months before the first diagnosis of PD (see Supplementary Table 2). Patient who never took anti-parkinsonian agents (such as levodopa, carbidopa, ropinirole hydrochloride, pramipexole dihydrochloride monohydrate, rotigotine, rasagiline or selegiline…etc.) after the first diagnosis of PD were also excluded. We also excluded the incomplete data, such as unknown sex or age. To clarify the relationship of developing dementia in PD, we excluded patients with a diagnosis of dementia (ICD-9-CM code 290, 331.0, 331.2, A210) before the first diagnosis of PD, or within 1 year of diagnosis of PD. The non-PD control subjects were selected randomly from the LHID 2005 and were matched with the ratio of 4:1 in the PD group for age, sex and the index date (the date of the first diagnosis of PD). Those who had a diagnosis of dementia (ICD-9-CM code 290, 331.0, 331.2, A210) before the index date and had a diagnosis of PD or secondary parkinsonism were excluded from the non-PD control group.

### Confounders

Because dementia is heterogeneous, it may coexist different types of pathologies and its risk factors are protean, including cardiovascular disease, cerebrovascular disease, metabolic and psychiatric factors, diet, lifestyle, and education (24, 25). We selected some well-known risk factors as our potential confounders, including hypertension, diabetes mellitus (DM), hyperlipidemia, chronic kidney disease (CKD) and ischemic heart disease (IHD). Hypertension, DM, hyperlipidemia and CKD were selected because they were potent factors for developing cerebrovascular disease. IHD or coronary artery disease was also suggested as a potential modified risk factor of dementia (26, 27).

### Outcome

**Figure 1:**
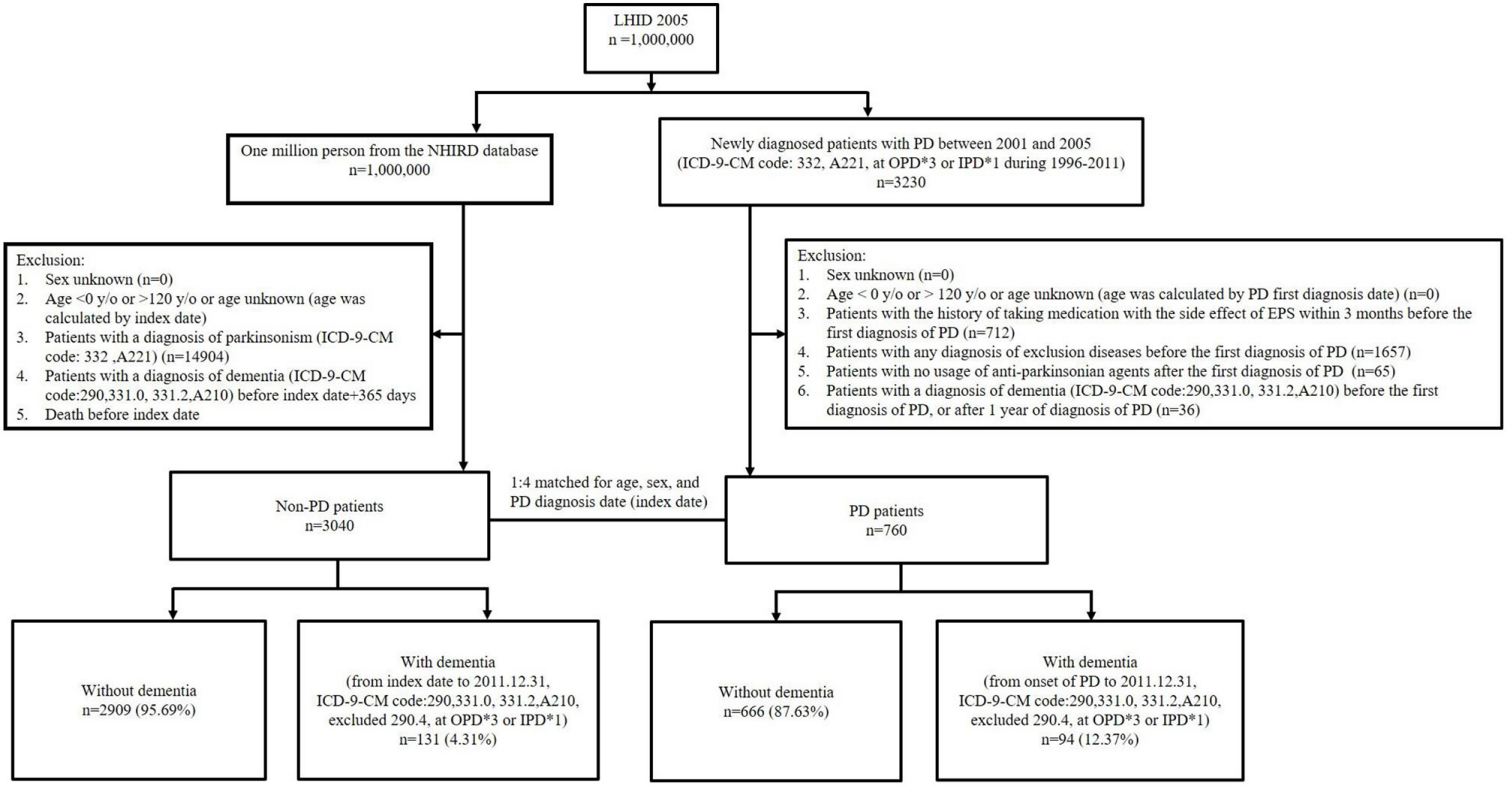
Flow diagram for study participants. LHID, Longitudinal Health Insurance Database; NHIRD, National Health Insurance Research Database; PD, Parkinson's disease; OPD, outpatient department; IPD, inpatient department; EPS, extrapyramidal symptoms.

The primary clinical outcome was the development of dementia. Both the PD and non-PD groups were followed up from the date of the first diagnosis of PD or the index date until December 31, 2011. We defined patients with the diagnosis of dementia by encoded with ICD-9-CM codes of 290, 331.0, 331.2, and A210. A previous study in Taiwan reported that the diagnostic accuracy of dementia is ~90% by using the definition of ICD-9-CM code (28). To increase the validity, we diagnosed patients with dementia when it was defined in three or more consecutive outpatient visits or in one or more inpatient settings. The types of dementia couldn't be clearly defined due to the database limitations. According to diagnosing with dementia or not, PD patients and non-PD control subjects were subsequently divided to four groups: PD with dementia, PD without dementia, non-PD with dementia and non-PD without dementia (see Figure 1). The secondary clinical outcome included the impact of different potential confounders on the risk of developing dementia.

### Statistics

For the PD and non-PD groups, we used the Student's *t*-test and Pearson chi-squared test to compare their age, sex, and confounders (hypertension, DM, hyperlipidemia, CKD and IHD) between them. We calculated the person-time incidence rate of dementia as the number of events divided by person-years from subjects in two groups. We compared the risk of dementia incidence between the PD and non-PD groups using Cox proportional hazards regression model and obtained the hazard ratio (HR) and 95% confidence intervals (CIs) after adjusting for age, sex, hypertension, DM, hyperlipidemia, CKD and IHD. We subsequently stratified the PD and non-PD groups by ages and sex. We used two different age stratifications: age group 1 was stratified by <50, 50–69, and ≥70 year-old, and age group 2 was stratified by <60, 60–69, and ≥70 year-old. The age stratifications were based on the major clinical phenotypes of PD (10, 11). The average onset age of the early-onset PD was around 50 years, and the older-onset PD was around 67 years. However, there were few patients <50 years old developing dementia in our cohort. We designed the age group 2 (<60, 60–69, >70 years old) for statistical analysis. We then calculated their adjusted HRs to explore the effects of PD on the risk of dementia within different groups. To analyze the impacts of different potential confounders on the development of dementia in our cohort, adjusted HRs were calculated separately for the following factors: ages, sex, PD, hypertension, DM, hyperlipidemia, CKD and IHD, using Cox proportional hazards regression model. We also estimated the dementia-free survival rate of both PD and non-PD group using the Kaplan–Meier curve. All statistical tests were two-sided, conducted at a significance level of 0.05, and reported using *p*-value and/or 95% CIs for HRs, which excluded the value 1.00 were statistically significant. All statistical analyses were performed with SAS software version 9.4 (SAS Institute, Cary, NC, USA).

This study followed the STrengthening the Reporting of OBservational studiesin Epidemiology (STROBE) statement (29) and used the checklist to ensure that the relevant aspects of study design was addressed (see Supplementary Table 3).

## Results

The flow diagram of study participant selection is shown in Figure 1. There were 760 patients with newly diagnosed PD who were enrolled, and 3,034 patients without PD comprised the control group. The baseline clinical characteristics in our study subjects with and without PD are shown in Table 1. There were more patients with hypertension and diabetes mellitus (DM) in the PD group compared to the non-PD group (hypertension, 35.0 vs. 29.3%, respectively, *p* = 0.002; DM, 16.4 vs. 12.5%, respectively, *p* = 0.004). There were no significant differences in age, sex, hyperlipidemia, chronic kidney disease (CKD), and ischemic heart disease (IHD) between the PD and non-PD groups.

**Table 1:** Baseline clinical characteristics of study subjects with and without PD.

**Variable**	**Total (*****n*** **= 3,800)**		**Non-PD (*****n*** **= 3,040)**		**PD (*****n*** **= 760)**		***P*** **-value**
	***n*** **(%)**		***n*** **(%)**		***n*** **(%)**		
Age, years (mean ± SD)	60.8 ± 17.6		60.8 ± 17.6		61.0 ± 17.6		0.798^†^
Age group 1							1.000
<50	1,010	(26.6)	808	(26.6)	202	(26.6)	
50–69	1,240	(32.6)	992	(32.6)	248	(32.6)	
≥70	1,550	(40.8)	1,240	(40.8)	310	(40.8)	
Age group 2							1.000
<60	1,475	(38.8)	1,180	(38.8)	295	(38.8)	
60–69	775	(20.4)	620	(20.4)	155	(20.4)	
≥70	1,550	(40.8)	1,240	(40.8)	310	(40.8)	
Sex							1.000
Female	1,785	(47.0)	1,428	(47.0)	357	(47.0)	
Male	2,015	(53.0)	1,612	(53.0)	403	(53.0)	
Hypertension							0.002
No	2,643	(69.6)	2,149	(70.7)	494	(65.0)	
Yes	1,157	(30.4)	891	(29.3)	266	(35.0)	
Diabetes							0.004
No	3,296	(86.7)	2,661	(87.5)	635	(83.6)	
Yes	504	(13.3)	379	(12.5)	125	(16.4)	
Hyperlipidemia							0.213
No	3,383	(89.0)	2,716	(89.3)	667	(87.8)	
Yes	417	(11.0)	324	(10.7)	93	(12.2)	
Chronic kidney disease						0.329
No	3,740	(98.4)	2,995	(98.5)	745	(98.0)	
Yes	60	(1.6)	45	(1.5)	15	(2.0)	
Ischemic heart disease							0.178
No	3,344	(88.0)	2,686	(88.4)	658	(86.6)	
Yes	456	(12.0)	354	(11.6)	102	(13.4)	

† *Student's t-test; chi-squared test for all other P-values. PD, Parkinson's disease*.

We calculated the incidence rate and hazard ratio (HR) for PD-D using a Cox proportional hazard regression model (Table 2). The overall incidence rate of dementia was 17.5 per 1,000 person-years in the PD group and 5.7 per 1,000 person-years in the non-PD group. When stratified by age, there was a trend where older patients had a higher incidence rate of dementia in both the PD and non-PD groups, especially in the >70 years stratification (39.7 and 13.1 per 1,000 person-years, respectively). In age group 1, three PD patients were diagnosed with dementia in the <50 years stratification, with an incidence rate of 1.8 per 1,000 person-years. No controls were diagnosed with dementia in this young age stratification. In age group 2, PD patients who were <60 years old also had a higher incidence rate of dementia (2.0 per 1,000 person-years) compared to controls (0.3 per 1,000 person-years). Both female and male patients had a higher incidence of dementia in the PD group. Female patients also seemed to have a higher incidence rate of dementia than male patients in both groups (19.6 vs. 15.6 per 1,000 person-years in the PD group and 6.1 vs. 5.3 per 1,000 person-years in the non-PD group, respectively).

**Table 2:** Incidence rate and hazard ratio of dementia associated with PD in Cox's regression analysis, as stratified by age or sex.

	**Non-PD (*****n*** **= 3,040)**			**PD (*****n*** **= 760)**			**Crude HR**	**(95% CI)**	***P*** **-value**	**Adjusted HR**	**(95% CI)**	***P*** **-value**
	**Event**	**Person-years**	**Incidence rate of dementia^†^**	**Event**	**Person-years**	**Incidence rate of dementia^†^**						
Overall	131	23,017	5.7	94	5,363	17.5	3.10	(2.38–4.04)	<0.001	3.23	(2.47–4.22)	<0.001
**Age group 1 (years)**												
<50	0	6,776	0.0	3	1,698	1.8	—	—	—	—	—	—
50–69	22	7,938	2.8	20	1,878	10.6	3.87	(2.11–7.10)	<0.001	3.80	(2.07–6.97)	<0.001
≥70	109	8,303	13.1	71	1,787	39.7	3.12	(2.31–4.21)	<0.001	3.15	(2.33–4.26)	<0.001
**Age group 2 (years)**												
<60	3	9,892	0.3	5	2,442	2.0	6.76	(1.62–28.3)	0.009	6.55	(1.56–27.48)	0.010
60–69	19	4,822	3.9	18	1,134	15.9	4.08	(2.14–7.77)	<0.001	4.04	(2.11–7.74)	<0.001
≥70	109	8,303	13.1	71	1,787	39.7	3.12	(2.31–4.21)	<0.001	3.15	(2.33–4.26)	<0.001
**Sex**												
Female	69	11,322	6.1	51	2,607	19.6	3.27	(2.28–4.69)	<0.001	3.39	(2.36–4.89)	<0.001
Male	62	11,695	5.3	43	2,756	15.6	2.93	(1.98–4.32)	<0.001	3.17	(2.14–4.69)	<0.001

† *per 1,000 person-years. Adjusted HR was adjusted for Age, sex, hypertension, diabetes, hyperlipidemia, chronic kidney disease, ischemic heart disease. PD, Parkinson's disease*.

After adjusting for the confounders (hypertension, DM, hyperlipidemia, CKD, and IHD), the PD group had a significantly higher overall risk of developing dementia compared to controls (adjusted HR 3.23, 95% CI 2.47–4.22, *p* < 0.001). The PD group also had a significantly higher risk of developing dementia in each age stratification (age group 1: 50–69 years, adjusted HR 3.80, 95% CI 2.07–6.97, *p* < 0.001; >70 years, adjusted HR 3.15, 95% CI 2.33–4.26, *p* < 0.001; age group 2: <60 years, adjusted HR 6.55, 95% CI 1.56–27.48, *p* = 0.010; 60–69 years, adjusted HR 4.08, 95% CI 2.14–7.77, *p* < 0.001; >70 years, adjusted HR 3.15, 95% CI 2.33–4.26, *p* < 0.001). Although the incidence rate for dementia was low (only 2.0 per 1,000 person-years) in PD patients < 60 years of age, the risk was 6.55-times higher in the young age stratification compared to the non-PD group. For sex, the PD group also had a significantly higher risk of dementia in both female (adjusted HR 3.39, 95% CI 2.36–4.89; *p* < 0.001) and male (adjusted HR 3.17, 95% CI 2.14–4.69; *p* < 0.001) patients.

To investigate the impact of different confounding factors on the risk of dementia, we calculated their HRs using a Cox proportional hazards regression model (Table 3). We found that old age, female sex, and PD were the three important variables that were associated with the higher risk of dementia in our cohort. Patients with PD had a high HR for developing dementia (adjusted HR 3.23, 95% CI 2.47–4.22; *p* < 0.001). For our patients, the older their age, the higher HR they had for developing dementia (adjusted HR 1.10, 95% CI 1.08–1.12; *p* < 0.001). Male patients had a significantly lower risk of dementia compared to female patients (adjusted HR 0.73, 95% CI 0.56–0.95; *p* = 0.019). There were no significant effects of hypertension, DM, hyperlipidemia, CKD, or IHD on the risk of dementia.

**Table 3:** Hazard ratio associated with dementia in Cox's regression analysis, as stratified by confounders.

**Variable**	**Adjusted HR**	**95% CI**	***P*** **-value**
Age, years	1.10	(1.08–1.12)	<0.001
**Sex**			
Female	1.00	—	—
Male	0.73	(0.56–0.95)	0.019
**PD**			
No	1.00	—	—
Yes	3.23	(2.47–4.22)	<0.001
**Hypertension**			
No	1.00	—	—
Yes	0.96	(0.73–1.27)	0.789
**Diabetes**			
No	1.00	—	—
Yes	1.36	(0.97–1.91)	0.078
**Hyperlipidemia**			
No	1.00	—	—
Yes	0.76	(0.50–1.15)	0.198
**Chronic kidney disease**			
No	1.00	—	—
Yes	0.67	(0.21–2.10)	0.493
**Ischemic heart disease**			
No	1.00	–	–
Yes	1.00	(0.70–1.42)	0.984

*Adjusted HR was adjusted for Age, sex, hypertension, diabetes, hyperlipidemia, chronic kidney disease, ischemic heart disease. PD, Parkinson's disease*.

**Figure 2:**
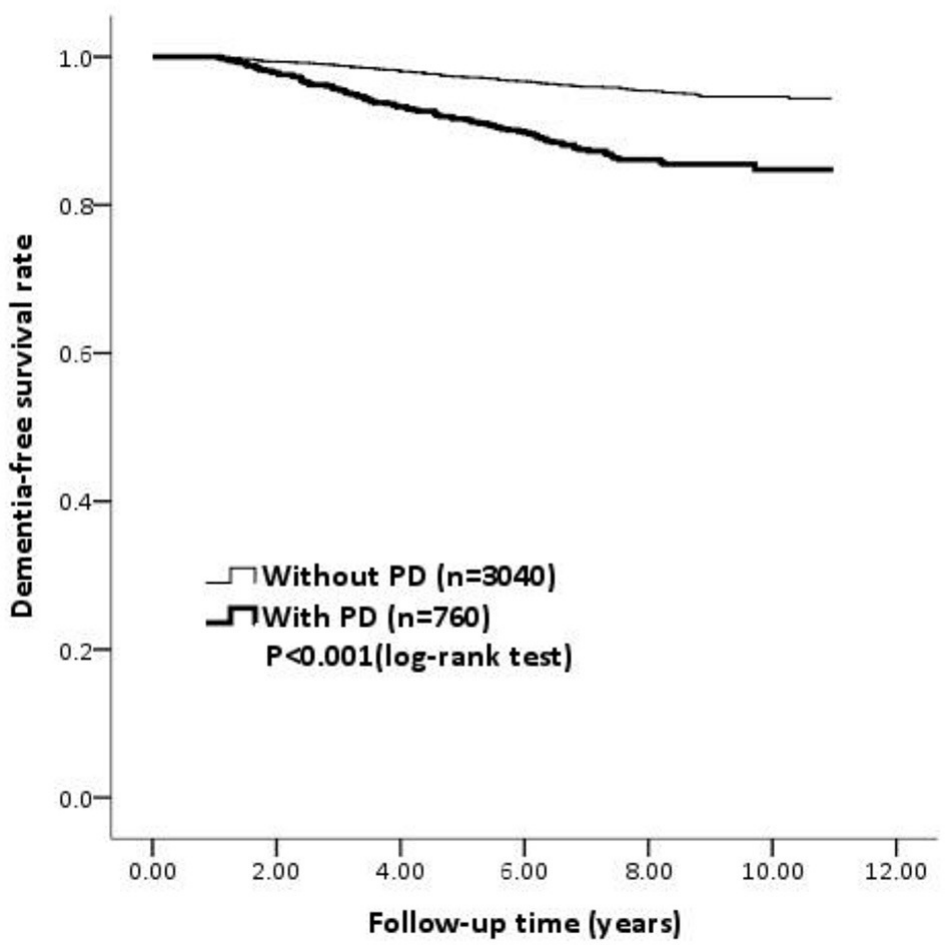
The survival curve determined using the Kaplan-Meier survival analysis for dementia among PD patients and non-PD patients. PD, Parkinson's disease.

Figure 2 shows the dementia-free survival rate of the PD and non-PD group using a Kaplan–Meier curve. There was a significantly lower dementia-free survival rate in patients with PD compared to the non-PD patients during the follow-up period (log-rank test, *p* < 0.001).

## Discussion

In our study, we investigated the incidence rate and the risk of dementia in patients with newly diagnosed PD. We used age-stratified methodology to analyze the risk of dementia in different age groups and to relate the different clinicopathological subtypes of PD. We speculated that older PD patients may result in a higher incidence rate of dementia because older people have a higher incidence of coexisting pathologies. Conversely, the progression of clinical cognitive impairment may be more consistent with the Braak PD staging in younger PD patients.

The incidence rates of dementia in PD have been investigated in several cohorts, and the range was about 30.0 to 112.5 per 1,000 patient-years (30). The incidence rate in our cohort was only 17.5 per 1,000 person-years in the PD group. The lower incidence rate might be explained by the relatively young age of our enrolled newly diagnosed PD patients (mean age, 61.0 ± 17.6 years) compared to other studies (mean age range, 63.7–76.7 years in different cohorts) (30). The overall risk of dementia was 3.23-times higher in the PD group than in the non-PD group, and this difference is consistent even in PD patients of different age stratifications or different sexes. Based on previous epidemiological studies, the risk of dementia in PD was reported to be 1.7 to 5.9-times higher compared to control groups (3, 4). Our results were also within this range. The HR was especially higher in the younger PD group (<60 years, adjusted HR 6.55, 95% CI 1.56–27.48, *p* = 0.010) compared to the older PD group (>70 years, adjusted HR 3.15, 95% CI 2.33–4.26, *p* < 0.001).

Halliday et al. described four major clinical phenotypes of PD with LPs based on the age of onset, dominant type of motor impairments, rate of progression, and presence of cognitive impairment (11). The four subtypes were early onset PD, tremor-dominant PD, postural instability and gait-dominant PD, and old-onset PD, respectively. The average onset age of the early-onset PD was around 50 years, and the older-onset PD was around 67 years. The rate of LP deposition and the co-existence of amyloid plaques correlated with different clinical phenotypes. Postural instability and gait-dominant PD and old onset PD subtypes had significantly more cortical Lewy bodies and amyloid-β plaques, which may lead to cognitive impairment, compared with tremor-dominant or younger onset subtypes.

In our study, the younger PD subgroup (<60 years old) had a lower incidence rate of dementia (2.0 per 1,000 person-years) compared to older PD patients (≥70 years old: 39.7 per 1,000 person-years), but the risk was 6.55-times higher than the controls of the same age. We speculated that the clinical progression in relatively earlier onset PD patients may correspond to the Braak PD staging. The rate of LP deposition is not rapid, and early onset PD patients have less cognitive impairment and a lower incidence rate of dementia. However, cortical LP is still the most important factor that is associated with PD-D, which is associated with a higher risk of dementia in earlier onset PD patients compared to controls of the same age.

However, the older PD group (>70 years old) had a significantly higher incidence rate of dementia (39.7 per 1,000 person-years) compare to controls. The higher incidence rate could not be explained only by the Braak PD staging of LP. In addition to LP, other co-pathologies may play important roles in the faster progression of cognitive impairment in older PD patients. Alzheimer-type (AD) pathologies (including amyloid-β deposition, diffuse and neuritic plaques, and neurofibrillary tangles) are particularly well-studied, but the role in PD-D remains controversial. Compta et al. quantitatively assessed the cortical pathologies in PD patients, and they showed that the combination of LP and AD pathologies most robustly correlated with PD-D (31). A synergistic interaction between the Alzheimer's disease-related protein aggregates and α-syn-containing inclusions has been proposed (32). The issue of synergistic effects of these two pathologies in PD-D requires further investigation. Other pathologies, such as cerebral amyloid angiopathy, hippocampal sclerosis, argyrophilic grains, and TAR DNA-binding protein 43, have been studied less and their roles remain unclear (10). We proposed that the higher incidence rate and risk of dementia in the older PD group resulted from cortical LP and other coincident pathologies, especially AD pathologies. Of note, cardiovascular risk factors, such as hypertension, diabetes mellitus and dyslipidemia, seemed to have less association with the risk of dementia in our cohort. We assumed that excluding patients with stroke initially may decrease the impact of these cardiovascular risks in developing dementia. By contrast, Parkinson's disease and age seemed to be more influential factors in our cohort.

Because the divergent pathological patterns of alpha-synuclein propagation in PD was yielded by later studies, the validity of Braak staging for all types of PD has been questioned (33). The staging showed more acceptable correlation in PD patients with early onset and prolonged duration with motor symptoms (34). Different routes of pathological progression and additional co-pathologies may contribute to different subtypes of PD. The clinicopathological features of Lewy body disease (LBD) are highly variable and heterogenous, and several neuropathological staging systems for Lewy body disease (LBD) was proposed. They included the Braak Lewy body stages, the Newcastle-McKeith criteria, the modified McKeith system by Leverenz et al. and the Unified Staging System by Beach and colleagues (35). These staging systems were based on the semi-quantitative scoring of Lewy bodies (LB) and Lewy neurites (LN) in defined subcortical and cortical areas. To enhance the inter-rater reliability and lower the frequency of non-classifiable cases, Lewy pathology consensus criteria (LPC) was proposed in 2021 (36). It used a dichotomized scoring of LB or LN (present or absent), and the diagnostic categories of olfactory-only, amygdala-predominant, brainstem, limbic, and neocortical LP. Of note, even low amounts of LP in neocortical areas may be categorized as neocortical LP. The category of neocortical LP according to the LPC criteria was associated with increasing odds of developing dementia in the multi-center study (36). It also implied that neocortical LP was one of the most important pathologies in LBD manifesting with dementia.

To the best of our knowledge, this is the first study to observe dementia associated with newly diagnosed PD in younger age stratifications. There was only one epidemiologic study that discussed the incidence rate of PD-D in Taiwan (37) and one study discussing the prevalence and risk factors of cognitive impairment in PD in Taiwan (38). The selected population of the studies was stratified by older age (> or >70 years old) and they were epidemiological studies. In our study, the aim was to analyze the risk of dementia in different age subgroups to reflect different clinicopathologic subgroups of PD using real-world evidence. To achieve our goal, we used younger age stratification and followed-up retrospectively. Although our study was only based on the epidemiologic data and there was no pathological support or evidence, we designed strict selection criteria to enroll newly diagnosed PD patients and to represent the real-world conditions for development of dementia in different age subgroups of PD patients. We linked the clinical observation to the pathogenesis of dementia in PD patients. The strength of our study was that we obtained these participants using national health insurance datasets to achieve a higher statistical power and to decrease the rate of patients who are lost to follow-up. We also conducted a risk analysis for the confounding factors of dementia using multivariate Cox proportional hazard regression.

There were some limitations in our study. First, our dataset had no information about baseline severity of motor or non-motor symptoms, relevant genetic factors or family history of genetic PD, educational level, clinical dominant types of motor symptoms, and presence of mild cognitive impairment (MCI). These unmeasured variables may affect the primary outcome of the newly diagnosed PD patients who were enrolled into our study. Second, coding errors are a known problem with the NHIRD, similar to all electronic health insurance databases in other countries. Nevertheless, the validation accuracy had been evaluated in other studies and reported a positive predictive value of more than 90% by using ICD-9-CM codes of PD in Taiwan (18–20). Additionally, the diagnosis of PD in our patients could not be assessed against the Movement Disorder Society (MDS) clinical criteria because of database limitations. To reduce bias as much as possible, our data selection process was cautious and excluded secondary or atypical parkinsonism. Third, the diagnosis of dementia in PD patients couldn't be appropriately assessed using the MDS criteria for PD-D, which may cause potential for bias in observational studies. Finally, despite the strict inclusion and exclusion criteria of our study, there may be surveillance bias of the time of patients diagnosed as dementia. Because about half of PD patients were under the care of neurologists in Taiwan (21), it could result in PD patients being diagnosed as dementia earlier and more frequently than controls.

In conclusion, we assumed that the progression of cognitive impairment may be consistent with the Braak PD staging in younger PD patients, and this led to a lower incidence rate of dementia compared to older PD patients but a higher risk of dementia compared with controls of the same age. However, older PD patients may have in a higher incidence rate of developing dementia because older patients have a higher incidence of coexisting pathologies, especially Alzheimer-type pathology. The cortical LP deposition and co-pathologies of the old-onset PD subtype may lead to faster clinical progression and a higher incidence of cognitive impairment than the early onset PD subtype, which is similar to the postural instability and gait-dominant PD subtype, and this has many more cortical Lewy bodies and amyloid-β plaques compared to the tremor-dominant PD subtype. The results of our study by using real-world data possibly implied that there were different pathogenesis and pathologies causing dementia in younger and older PD patients.

## Data Availability Statement

The datasets presented in this article are not readily available because the datasets were generated by the Taiwan's National Health Insurance Research Database, which was safeguarded and accessed by a strict application process. Requests to access the datasets should be directed to cmh50@ms10.hinet.net.

## Author Contributions

T-YC conceived the manuscript writing, study analysis, and interpretation. Y-HC and C-HL conceived the acquisition of data and statistical analysis. C-PY conceived the study concept design, acquisition of data, study analysis, and interpretation. M-HC conceived the study concept design, acquisition of data, study analysis and interpretation, and revision of manuscript. All authors contributed to the article and approved the submitted version.

## Conflict of Interest

The authors declare that the research was conducted in the absence of any commercial or financial relationships that could be construed as a potential conflict of interest.

## Publisher's Note

All claims expressed in this article are solely those of the authors and do not necessarily represent those of their affiliated organizations, or those of the publisher, the editors and the reviewers. Any product that may be evaluated in this article, or claim that may be made by its manufacturer, is not guaranteed or endorsed by the publisher.
