# Supplementary material for: Age-Stratified Risk of Dementia in Parkinson's Disease: A Nationwide, Population-Based, Retrospective Cohort Study in Taiwan
Source: Front Neurol. 2021 Dec 24;12:748096. doi: 10.3389/fneur.2021.748096 (PMC8740231; doi:10.3389/fneur.2021.748096)
Supplement: Supplementary file 1 [file Table_1.DOC]

**Supplementary table 1. Diseases with the risk of secondary or atypical Parki**nsonism

| **Diseases** | **ICD-9-CM** |
| --- | --- |
| Stroke | 430-438/A290-A294, A299 |
| Dementia | 290,331.0, 331.2/A210 |
| Meningitis, encephalitis | 00321, 0065, 0130-3, 0136, 0360-1, 0460-3, 047, 0490-1, 0520, 0530, 0543, 05472, 0550, 05601, 062-4, 0721-2, 09041-2, 0941-2, 09481-2, 09487, 09882, 10081, 11283, 1142, 11501, 11511, 11591, 1300, 1390, 320-6 |
| Head injury | 800-1, 803-4, 850-4 |
| Hydrocephalus | 742.3, 741.0, 331.3-4 |
| Brain tumor | 191, 192.0-1, 192.8-9, 194.3-4, 198.3, 237.0-1, 237.5-6, 237.9, 239.6-7, 2250, 2252, ﻿2273-4, ﻿22802 |
| Congenital or hereditary disorders | 2750-1, 3334, 334, 740 |
| Hypoxic encephalopathy | 348.1, 997.01, 639.8, 669.4, 768.7, 779.2 |

**Supplementary table 2. Drugs with the high risk of extrapyramidal symptoms**

| **Drug category** | **Generic drugs** |
| --- | --- |
| Anti-emetics | Metoclopramide, Prochlorperazine |
| Neuroleptics | Haloperidol, Amisulpride, Flupentixol, Fluphenazine, Levomepromazine, Pimozide, Thioridazine, Zuclopenthixol, Risperidone, Olanzapine, Aripiprazole |
| Calcium channel blocker | Flunarizine, Cinnarizine |
| Dopamine depleting agents | Reserpine, Tetrabenazine |
| Dopamine synthesis blocker | Methyldopa |

**Supplementary table 3. the STrengthening the Reporting of OBservational studiesin Epidemiology (STROBE) statement**—checklist of items that should be included in reports of observational studies

|  | | Item No. | Recommendation | Reported on Page No. |
| --- | --- | --- | --- | --- |
| **Title and abstract** | | 1 | (*a*) Indicate the study’s design with a commonly used term in the title or the abstract | 2 |
|  |  |  | (*b*) Provide in the abstract an informative and balanced summary of what was done and what was found | 2 |
| Introduction | | | |  |
| Background/rationale | | 2 | Explain the scientific background and rationale for the investigation being reported | 2 |
| Objectives | | 3 | State specific objectives, including any prespecified hypotheses | 3 |
| Methods | | | |  |
| Study design | | 4 | Present key elements of study design early in the paper | 4 |
| Setting | | 5 | Describe the setting, locations, and relevant dates, including periods of recruitment, exposure, follow-up, and data collection | 3 |
| Participants | | 6 | (*a*) Give the eligibility criteria, and the sources and methods of selection of participants. Describe methods of follow-up | 4 |
|  |  |  | (*b*) For matched studies, give matching criteria and number of exposed and unexposed | 4 |
| Variables | | 7 | Clearly define all outcomes, exposures, predictors, potential confounders, and effect modifiers. Give diagnostic criteria, if applicable | 4 |
| Data sources/ measurement | | 8 | For each variable of interest, give sources of data and details of methods of assessment (measurement). Describe comparability of assessment methods if there is more than one group | 3 |
| Bias | | 9 | Describe any efforts to address potential sources of bias | 4 |
| Study size | | 10 | Explain how the study size was arrived at | 4 |
| Quantitative variables | | 11 | Explain how quantitative variables were handled in the analyses. If applicable, describe which groupings were chosen and why | 4 |
| Statistical methods | | 12 | (*a*) Describe all statistical methods, including those used to control for confounding | 5 |
|  |  |  | (*b*) Describe any methods used to examine subgroups and interactions | N/A* |
|  |  |  | (*c*) Explain how missing data were addressed | N/A. There was no missing data. |
|  |  |  | (*d*) If applicable, explain how loss to follow-up was addressed | N/A |
|  |  |  | (*e*) Describe any sensitivity analyses | N/A |
| Results | | | |  |
| Participants | 13 | (a) Report numbers of individuals at each stage of study—eg numbers potentially eligible, examined for eligibility, confirmed eligible, included in the study, completing follow-up, and analysed | | 4 |
|  |  | (b) Give reasons for non-participation at each stage | | N/A |
|  |  | (c) Consider use of a flow diagram | | Figure 1. |
| Descriptive data | 14 | (a) Give characteristics of study participants (eg demographic, clinical, social) and information on exposures and potential confounders | | Table 1. |
|  |  | (b) Indicate number of participants with missing data for each variable of interest | | N/A |
|  |  | (c) Summarise follow-up time (eg, average and total amount) | | N/A |
| Outcome data | 15 | Report numbers of outcome events or summary measures over time | | Table 2. |
| Main results | 16 | (*a*) Give unadjusted estimates and, if applicable, confounder-adjusted estimates and their precision (eg, 95% confidence interval). Make clear which confounders were adjusted for and why they were included | | Table 2. |
|  |  | (*b*) Report category boundaries when continuous variables were categorized | | N/A. There were no continuous variables |
|  |  | (*c*) If relevant, consider translating estimates of relative risk into absolute risk for a meaningful time period | | N/A. There were no estimates of relative risk. |
| Other analyses | 17 | Report other analyses done—eg analyses of subgroups and interactions, and sensitivity analyses | | N/A |
| Discussion | | | |  |
| Key results | 18 | Summarise key results with reference to study objectives | | 6 |
| Limitations | 19 | Discuss limitations of the study, taking into account sources of potential bias or imprecision. Discuss both direction and magnitude of any potential bias | | 8 |
| Interpretation | 20 | Give a cautious overall interpretation of results considering objectives, limitations, multiplicity of analyses, results from similar studies, and other relevant evidence | | 7~8 |
| Generalisability | 21 | Discuss the generalisability (external validity) of the study results | | N/A |
| Other information | | | |  |
| Funding | 22 | Give the source of funding and the role of the funders for the present study and, if applicable, for the original study on which the present article is based | | There was no funding. |

* N/A= not available.

**Supplementary table 4. Use of anti-dementia drugs after the diagnosis of dementia*.**

|  | Non-PD with dementia (n=131) | | PD with dementia (n=94) | |
| --- | --- | --- | --- | --- |
|  | n (%) | | n (%) | |
| No | 113 | (86.3) | 88 | (93.6) |
| Cholinesterase inhibitors |  |  |  |  |
| Rivastigmine^#^ | 4 | (3.1) | 3 | (3.2) |
| Donepezil | 11 | (8.4) | 2 | (2.1) |
| Others |  |  |  |  |
| Memantine | 1 | (0.8) | 0 | (0.0) |
| More than two medications | 2 | (1.5) | 1 | (1.1) |

* The definition of use of anti-dementia drugs is at least one prescription after the diagnosis of dementia.

^#^ Rivastigmine was included in the scope of benefits for the treatment of PD with dementia by the Taiwan’s National Health Insurance since 2010.

PD=Parkinson’s disease.
